# Trajectories of adaptive and disturbed identity dimensions in adolescence: developmental associations with self-esteem, resilience, symptoms of depression, and borderline personality disorder features

**DOI:** 10.3389/fpsyt.2023.1125812

**Published:** 2023-04-24

**Authors:** Annabel Bogaerts, Laurence Claes, Koen Raymaekers, Tinne Buelens, Tim Bastiaens, Koen Luyckx

**Affiliations:** ^1^Department of Clinical Developmental Psychology, University of Amsterdam, Amsterdam, Netherlands; ^2^Faculty of Psychology and Educational Sciences, KU Leuven, Leuven, Belgium; ^3^Faculty of Medicine and Health Sciences, University Antwerp, Antwerp, Belgium; ^4^Fonds Wetenschappelijk Onderzoek, Brussels, Belgium; ^5^Department of Clinical Psychology, University of Amsterdam, Amsterdam, Netherlands; ^6^University Psychiatric Centre, KU Leuven, Kortenberg, Belgium; ^7^UNIBS, University of the Free State, Bloemfontein, South Africa

**Keywords:** identity, adolescence, self-esteem, resilience, depression, borderline

## Abstract

To advance our understanding of adolescents’ identity formation and how it may play into their psychological functioning, this study investigated developmental trajectory classes of adaptive and disturbed dimensions of identity formation, and whether adolescents belonging to different trajectory classes develop differently on self-esteem, resilience, symptoms of depression, and borderline personality disorder (BPD) features. Three-wave longitudinal data from 2,123 Flemish adolescents was used (54.2% girls; *M*_age_ = 14.64, range = 12–18 at T1). Results pointed to four trajectory classes of identity formation: adaptive identity, identity progression, identity regression, and diffused identity. The adaptive identity class presented with stable high levels of self-esteem and resilience, and stable low levels of symptoms of depression and BPD, whereas opposite results were obtained for the diffused identity class. The identity progression class reported an increase in self-esteem and resilience as well as a decrease in symptoms of depression and BPD, whereas opposite results were obtained for the identity regression class. These results emphasize that adaptive and disturbed dimensions of identity formation are closely related to markers of well-being and psychopathology among adolescents, and could help identify adolescents with an increased risk for negative psychological functioning or increased opportunity for positive psychological functioning.

## 1. Introduction

Identity formation is an essential developmental process in life, in which important changes commonly present at adolescence, a time when young individuals slowly transition from childhood to adulthood (1, 2). Identity formation has been increasingly linked to a wide array of indicators of psychosocial functioning and, more recently, to different psychiatric disorders, highlighting the transdiagnostic value and clinical relevance of identity functioning (3, 4). Nonetheless, research on identity development has particularly focused on normative identity processes in late adolescent samples, but has failed to chart *both* adaptive and disturbed dimensions of identity development in *early to late* adolescents. In addition, studies linking patterns of change in identity development to indicators of psychological functioning remain scarce. To address this gap, the present study investigated (1) developmental trajectories of adaptive and disturbed identity dimensions, (2) developmental trajectory classes of adaptive and disturbed identity dimensions, and (3) whether these classes develop differently on self-esteem, resilience, symptoms of depression, and borderline personality disorder (BPD) features in a large sample of early to late adolescents.

### 1.1. Adaptive and disturbed dimensions of identity formation in adolescence

Stimulated by physical, cognitive, social, and emotional maturation, young individuals entering adolescence generally feel inclined to rethink their childhood identifications and construct a more mature identity (2). Such a mature identity is expected to include a stable and personal set of goals, values, and beliefs, providing the individual with a sense of coherence and continuity, and guiding their future behavior and decision-making. For Marcia (5), a process of exploring different identity alternatives and then committing to one or more of these alternatives represents the most adaptive and effective way to develop such an identity (6). For many, this process may entail feelings of confusion, doubt, and discomfort, referred to as the identity crisis (2, 5). However, most adolescents are able to eventually work through this crisis and construct a personal identity that provides them with a sense of coherence and meaning (i.e., identity synthesis). Nevertheless, some adolescents may continue to struggle with contradictory feelings about their goals, values, and beliefs, and, often as a result, seem unable to settle on a personal identity [i.e., identity confusion; 2, 7].

Studies investigating the longitudinal development of a sense of identity synthesis or confusion in adolescence are scarce and have produced inconsistent findings. A recent three-wave longitudinal study indicated that young individuals’ sense of identity synthesis first decreased and then increased towards the end of adolescence, whereas their sense of identity confusion linearly increased throughout adolescence (8). Differently, results from a cohort-sequential study by Schwartz et al. (9) indicated no significant changes in identity synthesis and a linear decrease in identity confusion throughout adolescence. Still other results pointed to significant variability in young individuals’ sense of identity synthesis, but no significant changes in their sense of identity confusion during adolescence (10). The majority of research on identity development has studied changes in identity status or identity processes of exploration and commitment in late adolescent and (young) adult samples (see 11–13). Generally, this line of research suggested that most adolescents either indicate no change in identity status or identity processes, or increasingly develop a synthesized identity comprising stable identity commitments.

In addition to these variable-centered results, researchers have also adopted person-centered approaches to identify different developmental pathways of identity formation. Previous research on the development of a sense of identity in adolescence is limited to one study, which extracted five classes: a stable high synthesis and low confusion trajectory (20.4%), a stable low synthesis and high confusion trajectory (14.8%), a stable low synthesis and low confusion trajectory (4.7%), a high synthesis and high confusion with increases in synthesis and confusion trajectory (3.2%), and a moderate synthesis and moderate confusion with a slight increase in confusion trajectory (56.9%) (8). Whereas the first three classes seem to represent individuals who are, respectively, in a state of identity synthesis, identity confusion, or neither (possibly because they are (still) unconcerned with identity questions), the high synthesis and high confusion class may include adolescents who are actively working on their identity with strong conflict, whereas the moderate synthesis and confusion class may include adolescents who deal with a milder degree of conflict. Previous research on trajectory classes of identity exploration and commitment processes has been mostly carried out in emerging or young adults. For instance, Luyckx et al. (14) identified five trajectory classes among college students and employed individuals (i.e., achievement, foreclosure, moratorium, carefree diffusion, and troubled diffusion), each showing differential levels of and changes in exploration and commitment. Yet, recently, de Moor et al. (15) uncovered stable, progressive, and regressive classes of identity status change among early to late adolescents, reporting, respectively, no, positive, and negative changes in identity development throughout adolescence. Although current variable- and person-centered studies have generated important insights into how and when most adolescents develop a sense of identity, they remain predominantly focused on normative and behavioral processes involved in identity development, and fail to chart the development of potential clinical identity problems in community adolescents.

Attending to severe and potential clinical identity problems in adolescents seems important for several reasons. First, studies increasingly show that, in addition to developmentally-appropriate feelings of identity confusion, adolescents also struggle with identity problems such as lacking a sense of inner coherence, feeling a sense of discontinuity, and feeling broken or empty inside (16–19). Second, current dimensional classifications of psychopathology include problems related to self and identity as key constituent elements of personality pathology. In the DSM-5 Alternative Model for Personality Disorders, the presence and severity of personality pathology is determined by assessing disturbances in identity and self-direction (20). Sharp et al. (21) recently showed that impaired self and identity functioning is a significant indicator of BPD features in community-dwelling and clinically-referred adolescents. Third, studies in adolescents increasingly associate identity problems with a wide range of clinical disorders such as social–emotional disorders (3) and body-related disorders (e.g., eating disorders, body dissatisfaction, and non-suicidal self-injury; 19, 22, 23). Knowing how such identity problems develop may facilitate the identification (and, if necessary, treatment) of adolescents who deviate from more normative patterns of identity formation. But despite the transdiagnostic value and clinical relevance of identity, research investigating the development of clinical identity problems in adolescents is virtually absent.

As a way to advance such research and overcome the shortcomings of previous instruments that focus either on adaptive or disturbed dimensions of identity formation, scholars have developed instruments that are well suited to capture both adaptive and disturbed identity dimensions (17, 24). Kaufman et al. (24) constructed the Self-Concept and Identity Measure (SCIM), which was initially created to assess identity functioning in adults, but for which psychometric properties were established in adolescents later on (16). More so, the SCIM seems well suited to longitudinally assess identity development in adolescents (16). Starting from a developmental psychopathology perspective, the SCIM assesses identity in its healthy and disturbed dimensions. Individuals scoring high on *consolidated identity* reportedly experience a high degree of self-continuity, feel integrated and whole, and feel confident about who they are. These feelings are considered to be the result of having established stable identity commitments and self-defining roles, allowing individuals to navigate major life tasks (24). Individuals scoring high on *disturbed identity* reportedly experience a variety of identity problems including typical periods of uncertainty and more severe feelings of identity disturbance. Finally, clinical descriptions have alluded to an extremely maladaptive variant of identity, distinct from the more common presentation of a disturbed identity. This so-called *lack of identity* refers to feelings of inner emptiness, being broken, and feeling lost when thinking about who one is.

### 1.2. Identity formation and psychological functioning

Consistent with leading theories on psychosocial development (2, 25, 26), research has evidenced the close interconnectedness of identity and psychological well-being. For instance, adolescents who experience high levels of identity synthesis or who have made certain identity-related choices are more likely to experience high levels of life satisfaction (8), self-esteem (14, 27), and warm and supportive relationships (28) than adolescents who experience high levels of identity confusion or who have not yet enacted stable identity commitments.

Yet, a larger body of research has concentrated on how (problems in) identity formation relate to negative psychological functioning in adolescence as this life stage represents a critical time for the development of psychopathology (29). Generally, studies have demonstrated negative associations between identity synthesis and symptoms of depression, anxiety, eating disorders, and non-suicidal self-injury, as well as positive associations between identity confusion and these disorders (3, 23, 30). More so, as it is now increasingly assumed that features of personality disorders may already be present in adolescence (31–33), research on identity and (B)PD in adolescence is emerging. BPD is characterized by pervasive instability in affect, interpersonal relationships, and identity, and is marked by emotion dysregulation, impulsivity, and chronic feelings of emptiness (20). BPD is increasingly considered to be multifaceted, with different types having different developmental and neurobiological underpinnings (34, 35). So far, studies on identity and BPD in adolescents have evidenced that adaptive identity functioning is associated with low levels of (B)PD features, whereas disturbed identity functioning is associated with high levels of (B)PD features (16, 36). Furthermore, disturbed identity functioning and BPD features appear to become more closely associated with increasing age throughout adolescence (37). But as much as this line of research indicates the importance and clinical relevance of identity development, it does not allow us to determine which specific developmental patterns of identity formation are most closely related to both positive and negative psychological functioning throughout adolescence.

### 1.3. Hypotheses

The present study addressed three research objectives to increase our understanding of identity development and its associations with psychological functioning during adolescence. First, we investigated how both adaptive and disturbed identity dimensions (i.e., consolidated identity, disturbed identity, and lack of identity) develop across a time interval of 2 years in adolescent boys and girls. As previous research has obtained inconsistent findings regarding the development of normative identity dimensions (8, 10, 38) and there is a dearth of research on the development of clinically relevant identity dimensions, our hypotheses were based on leading identity theory (2, 7, 39). Generally, we expected to observe a linear increase in consolidated identity, as well as linear decreases in disturbed identity and/or lack of identity.

Second, we investigated whether we could identify different developmental trajectory classes of adaptive and disturbed identity dimensions in adolescent girls and boys. Building upon previous research (8, 9, 15), we tentatively expected to find classes characterized by (1) a stable high consolidated identity and stable low disturbed identity and/or lack of identity, (2) a stable low consolidated identity and stable high disturbed identity and/or lack of identity, (3) stable low identity dimensions, (4) stable high identity dimensions, and (5) respective increases and/or decreases in consolidated identity, and disturbed identity and/or lack of identity, or vice versa. However, as these studies (1) had a narrow focus on normative identity dimensions, (2) were carried out in adolescents being of different nationalities (i.e., Japanese, Hispanic, or Dutch nationalities), and (3) used different identity measures [i.e., the identity subscale from the Erikson Psychological Stage Inventory (EPSI) (7, 40) or the Utrecht-Management of Identity Commitments Scale (U-MICS) (41)], no definite hypotheses could be formulated.

Third, we investigated whether adolescents belonging to different trajectory classes developed differently on self-esteem, resilience (i.e., the ability to bounce back or recover from stress; 42). The text ‘symptoms of depression, and BPD features. Based on theory and previous research (2, 3, 14, 16, 26, 27, 37, 43), we hypothesized that classes with a stable high level of consolidated identity and stable low levels of disturbed identity and/or lack of identity would report high and/or increasing levels of self-esteem and resilience as well as low and/or decreasing levels of symptoms of depression and BPD. Opposite results were expected for classes with a stable low level of consolidated identity and stable high levels of disturbed identity and/or lack of identity. In addition, we hypothesized that classes with an increasing level of consolidated identity and decreasing levels of disturbed identity and/or lack of identity would report increasing levels of self-esteem and resilience, and decreasing levels of symptoms of depression and BPD, whereas opposite results were expected for classes with, respectively, decreasing and increasing levels of consolidated, disturbed and/or lack of identity.

As previous cross-sectional research has pointed to sex differences in mean scores on identity dimensions, symptoms of depression, BPD features (16, 30), and self-esteem (44), we conducted the primary analyses in the total group as well as in girls and boys separately. Specifically, as girls seem to be more prone to experience identity-related problems, we hypothesized to observe trajectory classes characterized by lower baseline levels of consolidated identity, as well as higher baseline levels of disturbed identity and/or lack of identity. In the absence of consistent previous research, no hypotheses on sex differences in slopes for identity dimensions could be formulated.

## 2. Materials and methods

### 2.1. Participants and procedure

The present study is part of the Longitudinal Identity research in Adolescence (LIA) project (45), a three-wave longitudinal study that was carried out in January 2018 (T1), January 2019 (T2), and January–February 2020 (T3). Data were collected in high school students recruited from eight secondary high schools in Flanders, the Dutch-speaking part of Belgium. Prior to data collection, school staff distributed an information letter and informed consent form among the students’ parents, as parental consent was required for participation of minor students. Data were collected during school hours in the presence of the researchers. In all schools, students completed the questionnaires using paper and pencil and were requested to hand in their completed questionnaires in a sealed envelope to one of the researchers. We administered two different versions of our survey to not overburden students and because they had to be able to finish the survey within one period. Whereas some questionnaires were administered to all participating students, some questionnaires were administered to approximately 25% or 75% of high school students. At all measurement points, students who graduated, switched to another school, or were absent on the day of data collection were contacted by letter and e-mail and were invited to complete the questionnaires online. Students who completed the questionnaires received a movie ticket as compensation. To ensure confidentiality and anonymity, and to match data across measurement points, students’ names were replaced by a unique code number. The present study was approved by the ethical committee of the Faculty of Psychology and Educational Sciences of KU Leuven.

At T1, a total of 3,483 high school students were contacted to participate in the LIA study. Of those who were contacted, a total of 2,313 students (66.4%) received active parental consent, of which 2,161 students actually agreed to participate in the study (response rate = 93.5%; 53.93% girls; *M*_age_ = 14.58, *SD* = 1.88, range = 10–21). At T2, a total of 1929 students participated (retention rate = 89.26%; 55.21% girls; *M*_age_ = 15.61, *SD* = 1.83, range = 11–22). Finally, T3 included a total of 1751 students (retention rate = 90.77%; 56.25% girls; *M*_age_ = 16.57, *SD* = 1.83, range = 12–23). For the present study, students younger than 12 and older than 18 at T1 were excluded from the sample as they were largely underrepresented. Eventually, this study included 2,123 students at T1 (54.2% girls; *M*_age_ = 14.64, *SD* = 1.81, range = 12–18), 1898 students at T2 (55.4% girls; *M*_age_ = 15.58, *SD* = 1.77, range = 13–19), and 1723 students at T3 (56.5% girls, *M*_age_ = 16.55, *SD* = 1.77, range = 14–20). At T1, 92.65% of the students self-reported being of Belgian nationality, 5.22% of Dutch nationality, 1.43% of another nationality, and for 0.7% information was missing. In Belgium, children generally start secondary education at age 12. The first 2 years (i.e., seventh and eighth grade), they all follow a general track. From the third year on, they can choose between general education, technical education, or art education, all of which prepare them for higher education (if desired). At T1, 34.6% of the students were in the seventh and eighth grade and followed the general track. The remaining students were in the ninth to twelfth grade and followed the general track (20.3%), the technical track (26.1%), or the arts track (19.1%) of secondary education. Finally, 68% of the students reported being part of an intact family, 20.1% reported their parents being divorced, 6.7% reported being part of a reconstituted family, 1.6% reported that one of their parents had deceased, and for 2.8% information was missing.

### 2.2. Measures

**Identity functioning**. All participants completed the Dutch translation of the Self-Concept and Identity Measure (SCIM; 24, 46) at all measurement points to assess adaptive and disturbed dimensions of identity formation. The SCIM consists of 27 self-report items measuring three subscales: consolidated identity (*n* = 10; e.g., *‘I always have a good sense about what is important to me’*), disturbed identity (*n* = 11; e.g., *‘The things that are most important to me change pretty often’*), and lack of identity (*n* = 6; e.g., *‘I feel empty inside, like a person without a soul’*). Items are rated on a 7-point Likert scale ranging from 1 (completely disagree) to 7 (completely agree). Although we administered the Dutch translation of the original 27-item SCIM, analyses of the present study were conducted using a 23-item version of the SCIM, which was previously validated among these Flemish adolescents (16). In this study, Cronbach’s alpha coefficients for consolidated identity, disturbed identity, and lack of identity were, respectively, 0.75, 0.82, and 0.92 at T1, 0.78, 0.84, and 0.92 at T2, and 0.79, 0.85, and 0.92 at T3.

**Self-esteem**. All students completed the Dutch version of the Rosenberg Self-Esteem Scale (RSES; 47) at all measurement points to assess their self-esteem. The RSES comprises 10 items measuring global self-esteem by assessing both positive and negative feelings about the self (e.g., *‘I feel that I have a number of good qualities’*). All items are rated on a 4-point Likert scale ranging from 1 (strongly disagree) to 4 (strongly agree). In the present study, Cronbach’s alpha coefficients for self-esteem were 0.89 at T1, 0.90 at T2, and 0.90 at T3.

**Resilience**. All students completed the Dutch translation of the Brief Resilience Scale (BRS-NL; 42, 48) at all measurement points. The BRS consists of 6 items, to be rated on a 5-point Likert scale ranging from 1 (completely disagree) to 5 (completely agree), which measure one’s perceived ability to bounce back or recover from stress (e.g.*, ‘It is hard for me to snap back when something bad happens’*). Previous research has demonstrated that the BRS produces valid and reliable scores among student samples (48). In the present study, Cronbach’s alpha coefficients for resilience were 0.88 at T1, 0.90 at T2, and 0.91 at T3.

**Symptoms of depression**. At every measurement point, symptoms of depression were assessed using the depression subscale of the Symptom Checklist-90 (SCL-90; 49). The SCL-90 is a self-report questionnaire developed to measure a broad range of mental and physical problems. The depression subscale consists of 16 items (e.g., *‘Feeling hopeless about the future’*). Students are asked to indicate to what extent the items reflect their feelings or behavior of the past week. All items are rated on a 5-point scale ranging from 1 (not at all) to 5 (extremely). The depression subscale of the SCL-90 appears to produce reliable and structurally valid test scores (50). In the present study, Cronbach’s alpha coefficients for depression were 0.93 at T1, 0.94 at T2, and 0.94 at T3.

**Borderline personality disorder features**. Close to 75% of our sample (*n* = 1,540 at T1) completed the Borderline Personality Features Scale for Children (BPFS-C; 51) at all measurement points. The BPFS-C consists of 11 self-report items to be rated on a scale from 1 (not true at all) to 5 (always true; e.g., *‘I go back and forth between different feelings, like being mad or sad or happy’*). In the present study, Cronbach’s alpha coefficients for borderline personality disorder features were 0.85 at T1, 0.86 at T2, and 0.85 at T3.

### 2.3. Statistical analyses

Preliminary analyses were conducted using IBM SPSS Statistics version 27. First, we computed descriptive statistics of all study variables (i.e., means, standard deviations, and minima and maxima) for the total group. Second, we investigated sex differences in study variables at T1 by conducting (1) a multivariate analysis of variance (MANOVA) with sex as a fixed factor and consolidated identity, disturbed identity, lack of identity, self-esteem, resilience, and symptoms of depression as dependent variables (*N* = 2,123), and (2) a univariate analysis of variance (ANOVA) with sex as a fixed factor and BPD features at T1 as a dependent variable (*N* = 1,540). If a significant Wilks’ Lambda (λ) was obtained for the MANOVA, Bonferroni corrected univariate post hoc tests (to adjust for multiple comparisons) were considered. Third, associations between study variables at T1 and age were investigated using Pearson correlations. Correlations were considered statistically significant at the *p* < 0.007 level after Bonferroni adjustment (i.e., the pre-specified level of significance, *p* = 0.05, was divided by the number of simultaneously tested hypotheses, which is 7; 52). Fourth, associations among study variables were investigated using Pearson correlations as well and were considered significant at the *p* < 0.002 level (i.e., *p* < 0.05 divided by 21) after Bonferroni adjustment.

Primary analyses were conducted in Mplus version 8.0 (53). Full Information Maximum Likelihood (FIML) estimation was used, which provides unbiased parameter estimates in case data are missing at random or missing completely at random (54, 55). Furthermore, as sample size calculations for Structural Equation Models (SEM) indicated that a sample size of 981 adolescents would allow us to detect effect sizes as small as 0.15 with a power of 0.80, developmental trajectories could be estimated within a SEM framework (56). First, we performed multivariate Latent Growth Curve Modeling (LGCM) to examine developmental trajectories of identity dimensions using maximum likelihood estimation with robust standard errors (MLR). LGCM is a variable-centered approach as it estimates intra-individual growth trajectories by specifying the mean and variance of two latent growth factors: intercept (or initial level) and slope (or rate of change; 57). As variables were assessed at three measurement points that were equally spaced in time (time intervals of 1 year), factor loadings of slopes were fixed to 0, 1, and 2 for T1, T2, and T3, respectively. Model fit was evaluated by means of the following four fit indices: (1) the Satorra-Bentler chi-square index (S-Bχ^2^), which should be as small as possible, (2) the Comparative Fit Index (CFI), which should exceed 0.90 and preferably 0.95 for excellent fit, (3) the Root Mean Square Error of Approximation (RMSEA), which should be below 0.08 and preferably below 0.05 for excellent fit, and (4) the Standardized Root Mean Square Residual (SRMR), which should be below 0.10 (58–60). To compare boys and girls with regard to their trajectories of identity dimensions, a multi-group analysis was conducted. In essence, we compared a multivariate LGC model in which the estimated growth parameters of the developmental trajectories could vary among boys and girls (i.e., an unconstrained model) to models in which these growth parameters were constrained to be equal across sex (i.e., constrained models). We considered two constrained models: (1) a model in which the intercepts were constrained to be equal across sex and (2) a model in which the slopes were considered to be equal across sex. To compare model fit of the unconstrained model to model fit of the constrained models, S-Bχ^2^ difference tests were considered (60).

Second, we performed multivariate Growth Mixture Modeling (GMM) for the total group and separately for boys and girls to identify trajectory classes of identity dimensions. GMM is a person-centered approach in which individuals are probabilistically assigned to latent classes based upon similar patterns of responses on specified variables (61). As GMM assumes individual growth trajectories to be heterogeneous within classes, the variance of intercept and slope within a class is freely estimated. Four criteria were used to determine the optimal number of classes (53): (1) the Bayesian Information Criterion (BIC) statistic for a solution with *k* classes should be lower than for a solution with *k-1* classes, (2) the Entropy (E) statistic, for which values should exceed 0.75 to indicate accurate classification (or high classification quality), as it represents the accuracy with which individuals are assigned to the classes based upon the posterior classification probabilities (62), (3) the bootstrapped Likelihood Ration Test (b-LRT), for which significant *p-*values indicate significantly improved model fit through including an additional class, and (4) proportions for the latent classes, which should cover at least 1% of the sample (63). Finally, to find a meaningful solution, class enumeration was ultimately determined by these fit indices in combination with theoretical justification, parsimony, and interpretability (64). After an accurate class solution was found, participants were assigned to the class for which their posterior probability of membership was highest.

Third, we performed multi-group LGCM to investigate whether individuals belonging to different trajectory classes developed differently on self-esteem, resilience, symptoms of depression, and BPD features. First, for each outcome variable separately, a fully unconstrained model was estimated in which intercept and slope could vary across classes. Model fit was evaluated by the S-Bχ^2^, CFI, RMSEA, and SRMR indices. Second, we estimated models in which intercepts were held equal across classes, followed by models in which slopes were held equal across classes. Using S-Bχ^2^ difference tests, we compared model fit of the unconstrained model to model fit of the two constrained models. If the fit of the constrained models was significantly poorer than the fit of the unconstrained model, this would indicate that classes differed significantly on intercept and/or slope. If significant differences were detected, intercepts and slopes were fixed in a pairwise manner across classes and S-Bχ^2^ difference tests were used to uncover which intercepts and/or slopes differed from one other.

## 3. Results

### 3.1. Preliminary analyses

Table 1 presents the descriptive statistics of study variables and sex differences in study variables. The MANOVA revealed significant sex differences at T1 (Wilks’ λ = 0.840, *F*(6, 2012) = 64.001, *p* < 0.001, partial η^2^ = 0.160). Specifically, and as detailed in Table 1, boys reported higher mean levels of consolidated identity, self-esteem, and resilience, as well as lower mean levels of disturbed identity, lack of identity, and symptoms of depression as compared to girls. The ANOVA indicated significant sex differences in BPD features at T1, with girls reporting higher mean levels of BPD features than boys. Associations between variables and age, and among variables at T1 are shown in Table 2. Consolidated identity and self-esteem were negatively associated with age, whereas lack of identity, symptoms of depression, and BPD features were positively associated with age. Disturbed identity and resilience were not significantly associated with age. Finally, at T1, consolidated identity was negatively associated with disturbed identity, lack of identity, symptoms of depression, and BPD features, and positively associated with self-esteem and resilience. Alternatively, disturbed identity and lack of identity were negatively associated with consolidated identity, self-esteem, and resilience, and positively associated with one another, symptoms of depression, and BPD features. The high zero-order correlations of lack of identity with symptoms of depression and BPD features could possibly be caused by overlap in the underlying construct that they intend to measure (e.g., they all allude to feelings of emptiness). However, removing items that expressed similar content across these measures did not meaningfully change the obtained correlations.

**Table 1:** Descriptive statistics and sex differences in study variables at Time 1.

	Total group		Girls	Boys	*F*	*df*	Partial η^2^
	*M (SD)*	Min – Max	*M (SD)*	*M (SD)*			
Consolidated identity	4.82 (0.98)	1.29–7.00	4.65 (1.00)	5.03 (0.90)	80.40***	1, 2017	0.038
Disturbed identity	3.04 (1.00)	1.00–6.80	3.18 (1.01)	2.88 (0.98)	44.93***	1, 2017	0.022
Lack of identity	2.38 (1.43)	1.00–7.00	2.72 (1.56)	1.99 (1.14)	140.07***	1, 2017	0.065
Self-esteem	2.87 (0.59)	1.00–4.00	2.72 (0.60)	3.06 (0.51)	178.46***	1, 2017	0.081
Resilience	3.16 (0.90)	1.00–5.00	2.86 (0.86)	3.52 (0.81)	309.55***	1, 2017	0.133
Symptoms of depression	1.85 (0.81)	1.00–5.00	2.09 (0.88)	1.57 (0.61)	221.43***	1, 2017	0.099
BPD features	1.55 (0.74)	0.00–3.82	1.73 (0.73)	1.30 (0.68)	137.51***	1, 1,528	0.083

*M* = mean; *SD* = standard deviation.

*** *p* < 0.001.

**Table 2:** Zero-order Pearson correlations between study variables and age, and among study variables at Time 1.

	Age	2	3	4	5	6	7
1. Consolidated identity	−0.07***	−0.50***	−0.64***	0.69***	0.43***	−0.56***	−0.52***
2. Disturbed identity	−0.01	-	0.62***	−0.56***	−0.42***	0.52***	0.65***
3. Lack of identity	0.15***		-	−0.76***	−0.53***	0.81***	0.74***
4. Self-esteem	−0.12***			-	0.53***	−0.72***	−0.69***
5. Resilience	−0.03				-	−0.55***	−0.55***
6. Symptoms of depression	0.16***					-	0.74***
7. BPD features	0.25***						-

*** *p* < 0.001.

### 3.2. Developmental trajectories of identity formation

The multivariate LGC model in the total group initially indicated unacceptable model fit (S-Bχ^2^(18) = 643.896, *p* < 0.001; CFI = 0.918; RMSEA = 0.128 with 90% CI [0.120–0.137]; SRMR = 036). After including two error correlations between identity dimensions at T2 (i.e., between consolidated identity and lack of identity, and between disturbed identity and lack of identity) as suggested by the modification indices, the multivariate LGC model had a good fit (S-Bχ^2^(16) = 141.205, *p* < 0.001; CFI = 0.984; RMSEA = 0.061 with 90% CI [0.052–0.070]; SRMR = 0.024). Table 3 presents means and variances of intercepts and slopes for all identity dimensions. Means and variances of all intercepts were significant, indicating substantial individual differences in identity dimensions at baseline. Mean slopes of disturbed identity and lack of identity were also significant, suggesting substantial change at a group level. More specifically, disturbed identity linearly decreased, whereas lack of identity linearly increased over time. The mean slope for consolidated identity was 0.02, *p* = 0.107, suggesting no significant linear change over time.

**Table 3:** The mean and variance of intercepts and slopes for identity dimensions.

	Total group		Girls		Boys	
Identity dimensions	Mean	Variance	Mean	Variance	Mean	Variance
Intercepts						
Consolidated identity	4.82***	0.72***	4.64***	0.76***	5.03***	0.58***
Disturbed identity	3.04***	0.76***	3.18***	0.79***	2.88***	0.67***
Lack of identity	2.40***	1.82***	2.74***	2.15***	1.99***	1.12***
Linear slopes						
Consolidated identity	0.02	0.14***	0.02	0.16***	0.02	0.11***
Disturbed identity	−0.06***	0.13***	−0.04**	0.14***	−0.07***	0.11***
Lack of identity	0.06***	0.37***	0.04	0.45***	0.07**	0.28***

** *p* < 0.01.

*** *p* < 0.001.

Regarding associations among intercepts of identity dimensions, the intercept of consolidated identity was negatively associated with the intercepts of disturbed identity (*r* = −0.49, *p* < 0.001) and lack of identity (*r* = −0.88, *p* < 0.001), whereas the intercepts of disturbed identity and lack of identity were positively associated (*r* = 0.87, *p* < 0.001). These correlations reflected the correlational pattern of identity dimensions at T1, presented in Table 2. Regarding associations among slopes of identity dimensions, the slope of consolidated identity was negatively related to the slopes of disturbed identity (*r* = −0.08, *p* < 0.001) and lack of identity (*r* = −0.18, *p* < 0.001), whereas the slopes of disturbed identity and lack of identity were positively associated with one another (*r* = 0.17, *p* < 0.001).

In comparing girls and boys with regard to their developmental trajectories of identity formation, model fit comparison between the unconstrained model [S-Bχ^2^(32) = 174.043, *p* < 0.001; CFI = 0.981; RMSEA = 0.065 with 90% CI [0.055–0.074]; SRMR = 0.027] and the constrained models in which intercepts [S-Bχ^2^(35) = 312.639, *p* < 0.001; CFI = 0.962; RMSEA = 0.086 with 90% CI [0.078–0.095]; SRMR = 0.083] or slopes [S-Bχ^2^(35) = 179.354, *p* < 0.001; CFI = 0.980; RMSEA = 0.062 with 90% CI [0.053–0.072]; SRMR = 0.028] were held equal across sex, indicated that the slopes could be fixed across sex [ΔS-Bχ^2^(3) = 3.431, *p* = 0.330]. Model fit was significantly worse when constraining intercepts across sex [ΔS-Bχ^2^(3) = 155.355, *p* < 0.001], as the intercept of consolidated identity was lower and the intercepts of disturbed identity and lack of identity higher in girls as compared to boys. Means and variances of intercepts and slopes for boys and girls separately can be found in Table 3.

### 3.3. Trajectory classes of identity formation

**Figure 1:**
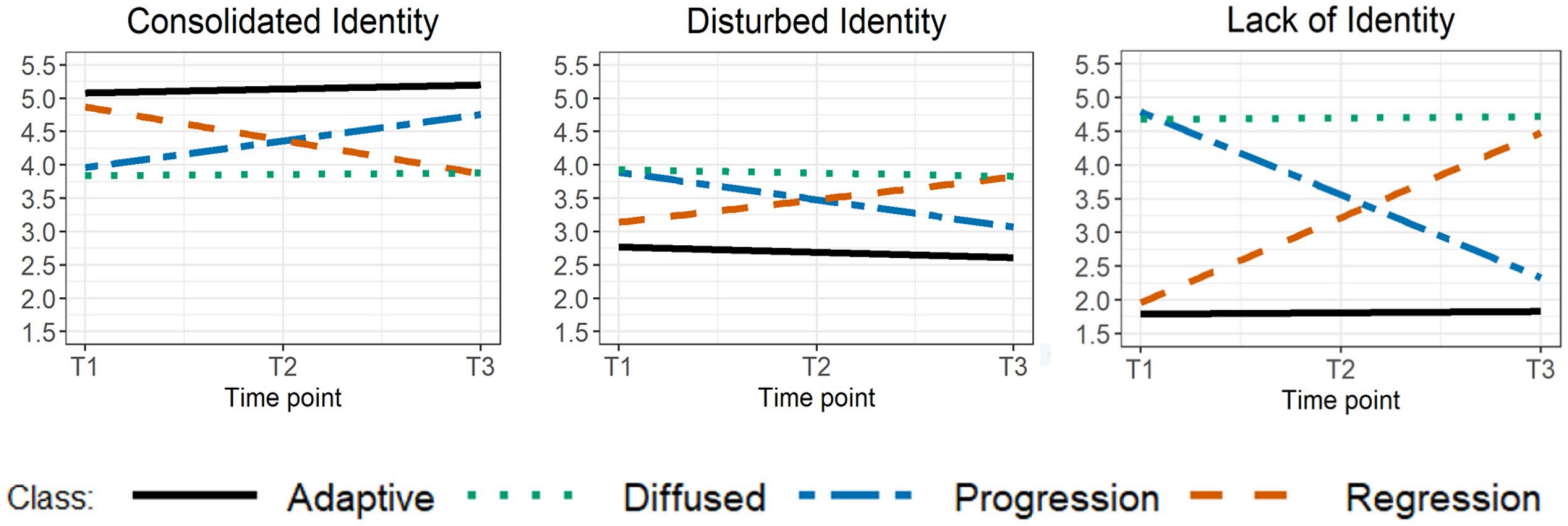
Mean intercepts and slopes for the identity dimensions in the four-class solution.

Table 4 presents the fit indices and trajectory class prevalence rates for GMM solutions with one through six classes for the total group. The 4-class solution was favored based on the fit indices and theoretical considerations. The first class (69.92%) was labeled *adaptive identity* as this class consisted of adolescents with a high initial level of consolidated identity, low initial levels of disturbed identity and lack of identity, a linear increase in consolidated identity, and a linear decrease in disturbed identity. The second class (7.83%) was labeled *identity progression* as individuals belonging to this class demonstrated a moderate initial level of consolidated identity, moderate to high initial levels of disturbed identity and lack of identity, a linear increase in consolidated identity, and linear decreases in disturbed identity and lack of identity. The third class (10.42%), labeled *identity regression,* consisted of adolescents with a high initial level of consolidated, a moderate initial level of disturbed identity, a low initial level of lack of identity, a linear decrease in consolidated identity, and linear increases in disturbed identity and lack of identity. The fourth class (11.83%) was labeled *diffused identity* as individuals belonging to this class chronically demonstrated moderate levels of consolidated identity and disturbed identity, and a high level of lack of identity. Mean intercepts and slopes for identity dimensions in the 4-class solution can be found in Table 5 and are visualized in Figure 1. We opted for the 4-class solution as the 5-class solution revealed classes of which some were merely slight variations of a similar class and did not add substantive meaning. For instance, the 5-class solution revealed two diffused identity-like classes. Both displayed stable moderate levels of consolidated identity (*M*_intercepts_ of 3.73 and 3.65), stable moderate to high levels of disturbed identity (*M*_intercepts_ of 3.95 and 4.01), and stable high levels of lack of identity (*M*_intercepts_ of 5.51 and 4.97) with small differences in intercept levels.

**Table 4:** Fit indices for different Growth Mixture Models for the total group, girls and boys.

				Trajectory class prevalence (%)					
Solution	BIC	E	b-LRT	1	2	3	4	5	6
Total group									
1	42827.080			100.00					
2	42188.118	0.88	*p* < 0.001	80.39	19.61				
3	41913.784	0.89	*p* < 0.001	70.11	20.37	9.52			
4	**41817.553**	**0.85**	***p* < 0.001**	**10.42**	**69.92**	**7.83**	**11.83**		
5	41727.440	0.83	*p* < 0.001	8.96	16.55	4.90	8.06	61.53	
6	41618.952	0.84	*p* < 0.001	8.96	5.52	11.27	8.30	4.20	61.76
Girls									
1	24205.562			100.00					
2	23960.477	0.84	*p* < 0.001	26.17	73.83				
3	23882.841	0.85	*p* < 0.001	62.52	12.70	24.83			
**4**	**23861.973**	**0.81**	***p* < 0.001**	**10.17**	**10.96**	**62.87**	**16.00**		
5	23844.529	0.82	*p* < 0.001	9.57	8.34	61.57	15.22	5.30	
6	23830.718	0.81	*p* < 0.001	10.78	4.61	8.87	54.52	7.48	13.74
Boys									
1	18380.382			100.00					
2	18059.291	0.92	*p* < 0.001	84.45	15.55				
3	17931.377	0.92	*p* < 0.001	17.30	77.03	5.66			
**4**	**17863.583**	**0.88**	***p* < 0.001**	**72.50**	**4.22**	**13.49**	**9.78**		
5	17781.020	0.89	*p* < 0.001	9.06	68.90	2.37	15.35	4.33	
6	17748.723	0.90	*p* < 0.001	2.27	3.50	2.47	15.14	8.24	68.38

The selected class-solutions are presented in bold.

**Table 5:** Mean intercepts (I) and slopes (S) for the four-class solution for the total group, girls, and boys.

	Total group		Girls		Boys	
	I	S	I	S	I	S
Class 1: Adaptive identity						
Consolidated identity	5.08	0.06***	4.96	0.05**	5.26	0.05*
Disturbed identity	2.77	−0.08***	2.86	−0.06**	2.62	−0.09**
Lack of identity	1.79	0.02	1.95	0.04	1.51	0.02
Class 2: Identity progression						
Consolidated identity	3.96	0.40***	3.85	0.42***	4.38	0.17*
Disturbed identity	3.89	−0.41***	3.91	−0.39***	3.57	−0.26***
Lack of identity	4.79	−1.23***	4.97	−1.20***	3.67	−0.59***
Class 3: Identity regression						
Consolidated identity	4.87	−0.50***	4.78	−0.51***	4.96	−0.46***
Disturbed identity	3.14	0.34***	3.16	0.36***	3.04	0.30***
Lack of identity	1.96	1.26***	2.02	1.34***	1.13	1.13***
Class 4: Diffused identity						
Consolidated identity	3.84	0.02	3.83	0.01	3.59	0.15
Disturbed identity	3.93	−0.05	3.94	−0.06	4.10	−0.01
Lack of identity	4.68	0.02	4.74	0.03	4.82	0.16

* *p* < 0.05.

** *p* < 0.001.

*** *p* < 0.001.

Girls and boys were unequally distributed across classes [χ^2^(3) = 111.61, *p* < 0.001]. Girls were underrepresented in the adaptive identity class (47%) and overrepresented in the identity progression (77%), identity regression (62%), and diffused identity (74%) classes. As LGCM pointed to significant differences in girls’ and boys’ levels of identity dimensions at baseline (see Table 3), GMM was also performed for girls and boys separately. The fit indices and trajectory class prevalence rates for one through six class-solutions for girls and boys are displayed in Table 4. Consistent with the total group, we selected the 4-class solution in girls and boys in which classes again represented adaptive identity, identity progression, identity regression, and diffused identity. The adaptive class represented 62.87% of girls and 75.50% of boys, the identity progression class represented 10.96% of girls and 13.49% of boys, the identity regression class represented 10.17% of girls and 9.78% of boys, and the diffused identity class represented 16% of girls and 4.22% boys. Mean intercepts and slopes for the identity dimensions in the 4-class solution in girls and boys can be found in Table 5.

### 3.4. Linking trajectory classes of identity formation to indicators of psychological functioning

Table 6 presents all parameter estimates of the multi-group LGCM of self-esteem, resilience, symptoms of depression, and BPD features in girls and boys. First, for self-esteem, the unconstrained model had a good fit in girls [S-Bχ^2^(4) = 9.464, *p* = 0.051; CFI = 0.992; RMSEA = 0.069 with 90% CI [0.000–0.127]; SRMR = 0.017] and an excellent fit in boys [S-Bχ^2^(4) = 6.914, *p* = 0.141; CFI = 0.996; RMSEA = 0.055 with 90% CI [0.000–0.122]; SRMR = 0.021]. Model fit comparison indicated that constraining the intercepts as equal across classes was not allowed in girls [ΔS-Bχ^2^(3) = 392.184, *p* < 0.001] or boys [ΔS-Bχ^2^(3) = 184.668, *p* < 0.001]. Similarly, constraining the slopes as equal across classes was not allowed in girls [ΔS-Bχ^2^(3) = 264.071, *p* < 0.001] or boys [ΔS-Bχ^2^(3) = 148.151, *p* < 0.001]. In girls, follow-up analyses indicated that all pairs of intercepts differed from one another, except for the intercepts of identity progression and diffused identity classes [ΔS-Bχ^2^(1) = 1.287, *p* = 0.257], in which girls reported the lowest levels of self-esteem at baseline. Girls in adaptive identity and identity regression classes reported higher levels of self-esteem at baseline, with girls in the adaptive identity class reporting the highest self-esteem. Furthermore, in girls, all pairs of slopes differed from one another, except for the slopes of adaptive identity and diffused identity classes [ΔS-Bχ^2^(1) = 1.064, *p* = 0.302], in which girls demonstrated relatively stable levels of self-esteem. Differently, girls in the identity progression class seemed to increase in self-esteem, whereas girls in the identity regression class seemed to decrease in self-esteem. In boys, follow-up analyses indicated that all pairs of intercepts and slopes differed from one another.

**Table 6:** Baseline parameter estimates of multi-group Latent Growth Curve Modeling in girls and boys.

	Trajectory classes of identity dimensions			
Parameters	Adaptive identity	Identity progression	Identity regression	Diffused identity
**Girls**				
Self-esteem				
*M*_intercept_	2.95^a^	2.12^b^	2.84^c^	2.07^b^
*M*_slope_	−0.02^a^*	0.29^b^***	−0.36^c^***	0.00^a^
Resilience				
*M*_intercept_	3.05^a^	2.25^b^	3.02^a^	2.38^b^
*M*_slope_	−0.02^a^	0.28^b^***	−0.34^c^***	−0.04^a^
Symptoms of depression				
*M*_intercept_	1.71^a^	3.13^b^	1.80^a^	3.07^b^
*M*_slope_	0.05^a^**	−0.50^b^***	0.63^c^***	0.05^a^
BPD features				
*M*_intercept_	1.42^a^	2.46^b^	1.58^c^	2.40^b^
*M*_slope_	0.04^a^**	−0.25^b^***	0.40^c^***	0.04^a^
**Boys**				
Self-esteem				
*M*_intercept_	3.20^a^	2.56^b^	3.03^c^	2.15^d^
*M*_slope_	0.02^a^*	0.16^b^***	−0.30^c^***	−0.08^d^
Resilience				
*M*_intercept_	3.67^a^	3.07^b^	3.45^c^	2.68^d^
*M*_slope_	0.05^a^**	0.13^a^**	−0.29^b^***	0.01^a^
Symptoms of depression				
*M*_intercept_	1.37^a^	2.25^b^	1.63^c^	2.77^d^
*M*_slope_	0.00^a^	−0.23^b^***	0.42^c^***	0.13^a^
BPD features				
*M*_intercept_	1.06^a^	2.03^b^	1.42^c^	2.24^b^
*M*_slope_	0.00^a^	−0.11^b^**	0.30^c^***	0.06^a^

Trajectory classes with a different superscript significantly differ from one another.

* *p* < .05.

** *p* < .01.

*** *p* < 0.001.

Second, for resilience, the unconstrained model had an excellent fit in girls [S-Bχ^2^(4) = 7.149, *p* = 0.128; CFI = 0.995; RMSEA = 0.052 with 90% CI [0.000–0.113]; SRMR = 0.017] and boys [S-Bχ^2^(4) = 7.029, *p* = 0.134; CFI = 0.993; RMSEA = 0.056 with 90% CI [0.000–0.123]; SRMR = 0.028]. Constraining the intercepts as equal across classes was again not allowed in girls [ΔS-Bχ^2^(3) = 133.188, *p* < 0.001] or boys [ΔS-Bχ^2^(3) = 80.081, *p* < 0.001]. Similarly, constraining the slopes to be equal across classes significantly worsened model fit in girls [ΔS-Bχ^2^(3) = 77.290, *p* < 0.001] and boys [ΔS-Bχ^2^(3) = 50.272, *p* < 0.001]. In girls, only adaptive identity and identity regression classes [ΔS-Bχ^2^(1) = 0.092, *p* = 0.762], and identity progression and diffused identity classes [ΔS-Bχ^2^(1) = 2.075, *p* = 0.150] did not differ from one another regarding initial levels of resilience. Furthermore, all pairs of slopes differed from one another, except for the slopes of adaptive identity and diffused identity classes [ΔS-Bχ^2^(1) = 0.312, *p* = 0.577], in which girls demonstrated, respectively, stable high and low levels of resilience over time. Girls in the identity progression class seemed to increase in resilience, whereas girls in the identity regression class seemed to decrease in resilience over time. In boys, all classes differed regarding level of resilience at baseline. Regarding slopes, only the identity regression class significantly differed from other classes with regard to the rate of change in resilience, as boys in this class seemed to decrease more in resilience over time as compared to boys in other classes.

Third, for symptoms of depression, the unconstrained model had an excellent fit in girls [S-Bχ^2^(4) = 4.504, *p* = 0.342; CFI = 0.999; RMSEA = 0.021 with 90% CI [0.000–0.094]; SRMR = 0.017] and boys [S-Bχ^2^(4) = 2.820, *p* = 0.588; CFI = 1.00; RMSEA = 0.000 with 90% CI [0.000–0.083]; SRMR = 0.013]. Constraining the intercepts was not allowed in girls [ΔS-Bχ^2^(3) = 373.746, *p* < 0.001] or boys [ΔS-Bχ^2^(3) = 185.213, *p* < 0.001]. Furthermore, constraining the slopes was not allowed in girls [ΔS-Bχ^2^(3) = 206.574, *p* < 0.001] or boys [ΔS-Bχ^2^(3) = 104.017, *p* < 0.001]. Once again, in girls, only adaptive identity and identity regression classes [ΔS-Bχ^2^(1) = 2.70, *p* = 0.132], and identity progression and diffused identity classes [ΔS-Bχ^2^(1) = 0.435, *p* = 0.510] did not differ regarding depressive symptoms at baseline. In boys, all classes differed regarding depressive symptoms at baseline. In girls and boys, all pairs of slopes differed from one another, except for the slopes of adaptive identity and diffused identity classes [girls: ΔS-Bχ^2^(1) = 0.039, *p* = 0.843; boys: ΔS-Bχ^2^(1) = 1.474, *p* = 0.225], in which adolescents showed, respectively, stable low and high levels of depressive symptoms over time.

**Figure 2:**
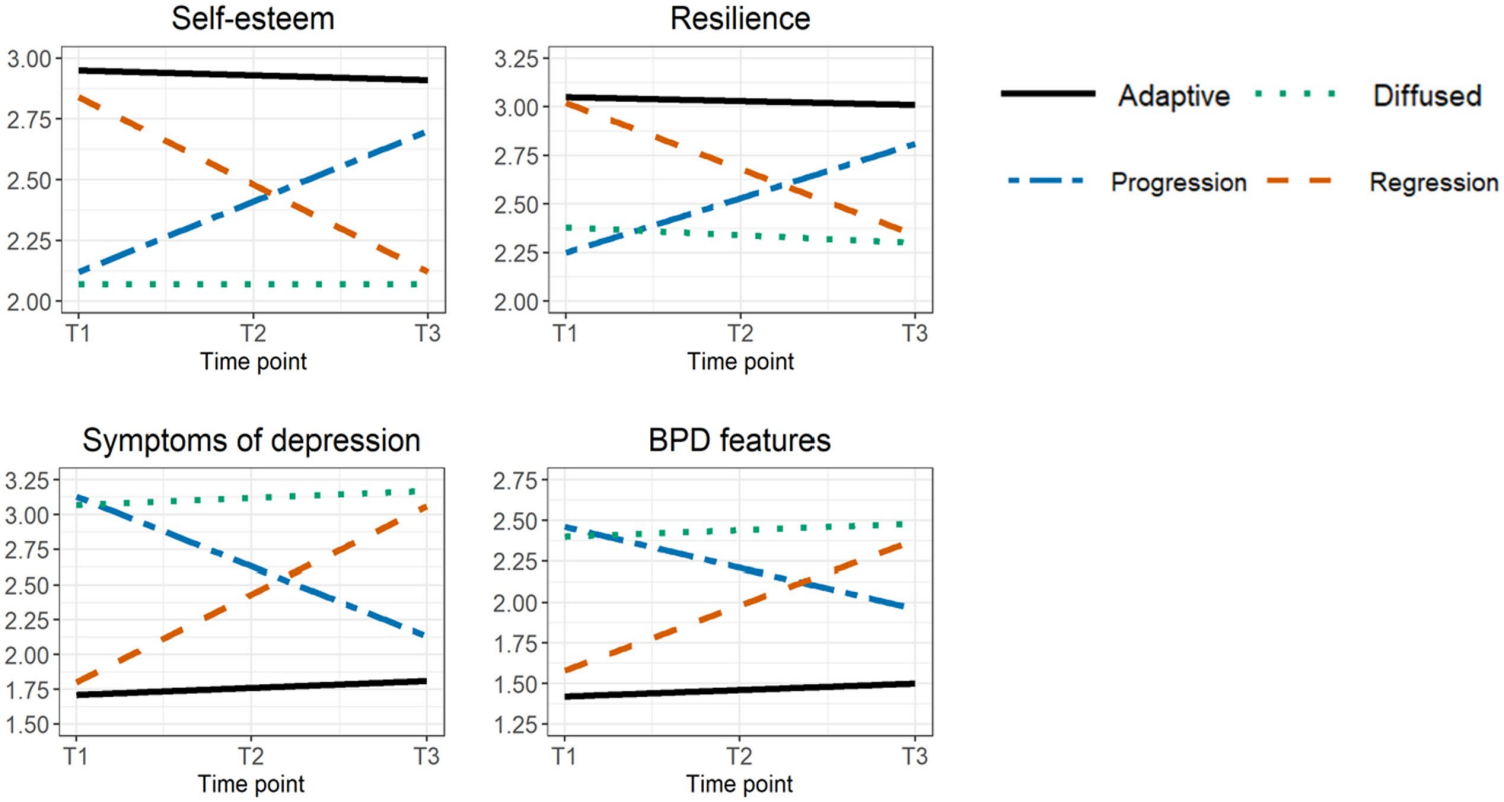
Parameter estimates of self-esteem, resilience, symptoms of depression, and BPD features for the four-class solution in girls.

Lastly, for BPD features, the unconstrained model had an excellent fit in girls [S-Bχ^2^(4) = 2.714, *p* = 0.607; CFI = 1.00; RMSEA = 0.000 with 90% CI [0.000–0.084]; SRMR = 0.013] and boys [S-Bχ^2^(4) = 1.343, *p* = 0.854; CFI = 1.00; RMSEA = 0.000 with 90% CI [0.000–0.065]; SRMR = 0.011]. Constraining the intercepts as equal across classes significantly worsened model fit in girls [ΔS-Bχ^2^(3) = 365.511, *p* < 0.001] and boys [ΔS-Bχ^2^(3) = 201.719, *p* < 0.001]. Constraining slopes as equal was also not allowed in girls [ΔS-Bχ^2^(3) = 138.962, *p* < 0.001] or boys [ΔS-Bχ^2^(3) = 35.206, *p* < 0.001]. In girls as well as in boys, only identity progression and diffused identity classes did not differ from one another regarding BPD features at baseline [girls: ΔS-Bχ^2^(1) = 1.145, *p* = 0.285; boys: ΔS-Bχ^2^(1) = 3.808, *p* = 0.051]. In girls and boys, all pairs of slopes differed from one another, except for the slopes of adaptive identity and disturbed identity classes [girls: ΔS-Bχ^2^(1) = 0.003, *p* = 0.953; boys: ΔS-Bχ^2^(1) = 1.013, *p* = 0.314]. Figure 2 displays all parameter estimates of the multi-group LGCM of self-esteem, resilience, symptoms of depression, and BPD features in girls, since results for girls and boys largely matched. As only 75% of the sample completed the BPFS-C, we repeated GMM for this subgroup and for girls and boys separately, as they appeared to be unequally distributed across classes [χ^2^(3) = 75.427, *p* < 0.001]. In the total subgroup and in the subgroups of girls and boys, the four-class solution was favored and included adaptive identity, identity progression, identity regression, and diffused identity classes (see Supplementary Table 1).

## 4. Discussion

The present study examined developmental trajectories and trajectory classes of adaptive and disturbed identity dimensions in adolescence, and examined how these trajectory classes were associated with baseline levels of and changes in self-esteem, resilience, symptoms of depression, and BPD features using three-wave longitudinal data from 2,123 adolescents aged 12 to 18 at T1.

With regard to the first study aim, LGCM pointed to no significant linear change in consolidated identity over a period of 2 years. The observed mean scores across the three measurement points indicated no significant changes in consolidated identity over time (*M*_T1_ = 4.824; *M*_T2_ = 4.805; *M*_T3_ = 4.868). Consistent with findings of Schwartz et al. (9) and Bogaerts et al. (65), this finding seems to suggest that adolescents’ sense of identity consolidation remains relatively stable over time. However, our finding differs from studies showing increases and/or decreases in identity synthesis from early to late adolescence (8, 10, 30). The difference in results could be ascribed to the three-wave longitudinal design of our study, which only allowed us to investigate linear change across three measurement points instead of age. For the present study, we considered developmental changes in consolidated identity in a large sample of 12 to 18-year-old adolescents without taking into account potential differences in identity formation during early, mid-, and late adolescence. For instance, Hatano et al. (66) demonstrated that young individuals’ sense of identity synthesis linearly decreased in early adolescence, whereas it linearly increased in mid- and late adolescence.

Furthermore, LGCM indicated a linear decrease in disturbed identity over a period of 2 years, which seems to suggest that identity-related problems such as sustained confusion about one’s identity and the tendency to mimic the values, beliefs, and goals of others in an attempt to acquire a sense of inner coherence decrease throughout adolescence (24, 43, 67). Our finding maps well onto previous research showing that youngsters tend to increasingly commit to and identify with their identity-related choices, whereas they engage less in reconsidering their choices and ruminating about them (68–70). Nonetheless, in contrast with our finding, recent studies have also pointed to increases in identity confusion and identity distress from early to late adolescence (8, 30). Authors have framed these findings within the notion of emerging adulthood, arguing that, for adolescents living in industrialized societies, settling into long-term adult roles is now delayed and has given way to an extended period of identity exploration, which may trigger identity confusion (71). The inconsistency in findings may be ascribed to variations in the operationalization of identity problems. Previous studies have used the EPSI (7, 40), in which identity confusion represents a sense of purposelessness, being without direction, and feeling unable to commit to and/or maintain commitments to life alternatives. Differently, the present study used the SCIM (24), in which disturbed identity represents both a sense of incoherence and instability as well as a strong dependence on others for providing a sense of coherence and guiding future behavior and decision-making. Thus, whereas the EPSI almost exclusively assesses individuals’ sense of personal identity, the SCIM assesses elements of both personal and social identity (i.e., the self in social situations). Previous research has demonstrated that social identity effects appear to be strongest in early adolescence as these young individuals are largely preoccupied with establishing a sense of belongingness and affiliation, and decrease throughout mid- and late adolescence (72, 73).

Finally, and rather unexpectedly at first, LGCM indicated a linear increase in lack of identity, a less common and pathological variant of identity dysfunctioning, which comes with feelings of inner emptiness and fragmentation. Although these feelings have been mainly considered within individuals with BPD or psychotic disorders (74–76), recent work shows that a lack of identity and feelings of inner emptiness are also experienced by individuals without mental health diagnoses (77, 78). A study among college students found that almost one in five students experience emptiness (79), and a recent study by Martin and Levy (80) indicated that feelings of emptiness were consistently endorsed by 10% of 22,217 US college students and significantly increased in women. As our study shows that experiencing a sense of inner emptiness and lack of identity is relatively common in adolescence and may even increase over time, research on the (pathological) nature of these feelings and how they diverge from other, highly researched identity problems (e.g., identity confusion and distress, and ruminative identity exploration) is recommended.

In addition to these developmental trends in the total group, multi-group LGCM indicated that girls and boys significantly differed regarding their levels of identity dimensions at baseline, although they showed similar rates of change in identity dimensions over time. Specifically, girls reported lower levels of consolidated identity as well as higher levels of disturbed identity and lack of identity at baseline as compared to boys. These findings align well with previous research showing less desirable identity functioning for adolescent girls than for adolescent boys (30, 65, 81).

With regard to the second study aim, GMM in the total group as well as in girls and boys revealed four trajectory classes of identity formation based on three dimensions capturing both adaptive and disturbed aspects of identity formation. First, the *adaptive identity class* (± 70%) presented with a high baseline and increasing level of consolidated identity, and low to moderate baseline and decreasing levels of disturbed identity and lack of identity. Hence, this class seems to represent a state of identity consolidation or identity synthesis, previously described by Erikson (2) as the hallmark of identity development and consistently found in previous research (31, 82, 83). In addition, these results emphasize the importance of attending to both relatively common identity problems (as captured by the disturbed identity scale) and more severe identity problems (as captured by the lack of identity scale) as adolescents in this class showed different baseline levels of and changes in disturbed identity and lack of identity. Specifically, they reported a low to moderate baseline and significantly decreasing level of disturbed identity, but a stable low level of lack of identity. Second, the *diffused identity class* (± 12%) showed stable moderate levels of consolidated identity and disturbed identity, and a stable high level of lack of identity over time. This class seems to represent a state of identity diffusion, an identity profile in which adolescents struggle with enduring feelings of emptiness and incoherence, which they attempt to compensate by anchoring their sense of identity in others. Although previous studies have described less desirable identity profiles in adolescence such as those characterized by high levels of identity confusion or rumination about identity alternatives [e.g., (8, 82, 83)], this study is among the first to demonstrate the occurrence of an identity profile characterized by more severe identity issues. Feelings of inner emptiness and fragmentation are hypothesized to go beyond developmentally-appropriate or more typical identity problems (24) and have been associated with a host of psychological disorders (16, 24, 84).

Third, the *identity progression class* (± 8%) was characterized by a moderate baseline and increasing level of consolidated identity, and moderate to high baseline and decreasing levels of disturbed identity and lack of identity. Fourth, the *identity regression class* (± 10%) demonstrated a high baseline and decreasing level of consolidated identity as well as low to moderate baseline and increasing levels of disturbed identity and lack of identity. Similarly, Schwartz et al. (9) found classes in which adolescents decreased or increased in identity confusion over time. Furthermore, identity status research in adolescents has pointed to regressive, but particularly progressive shifts in identity development (see 11–13). In line with our results, a recent study by de Moor et al. (15) distinguished between stable, progressive, and regressive identity classes, illustrating, respectively, no, positive, or negative identity cluster change over time. Our hypothesis was thus partially confirmed, as we identified adaptive and diffused identity classes (8), and also uncovered two developmental profiles of identity progression and regression. Furthermore, in line with previous research (38, 85), girls and boys were unequally distributed across trajectory classes. Girls were underrepresented in the adaptive identity class as well as overrepresented in the other classes. More so, GMM performed separately for girls and boys indicated that 63% and 16% of girls, and 76% and 4% of boys belonged to, respectively, adaptive identity and diffused identity classes.

With regard to the third study aim, multi-group LGCM results indicated that the identified trajectory classes manifested different baseline levels of and changes in self-esteem, resilience, symptoms of depression, and BPD features over time. In accordance with our hypotheses, theory, and previous research (3, 16, 27, 37, 82), adolescents in the adaptive identity class reported high levels of self-esteem and resilience, and low levels of depressive symptoms and BPD features. In contrast, adolescents in the diffused identity class reported low levels of self-esteem and resilience, and high levels of depressive symptoms and BPD features over time. Similarly, a study by Campbell et al. (86) among adolescents from community and clinical settings demonstrated that youth reporting the highest levels of identity confusion, identity disturbance, and feelings of emptiness were nearly twice as likely to report BPD features. Further along these lines, a study by Hatano et al. (66) showed that adolescents in high identity synthesis trajectories demonstrated significantly more life satisfaction than adolescents in high identity confusion trajectories.

Furthermore, adolescents in the identity progression class reported low baseline levels of self-esteem and resilience, and high baseline levels of symptoms of depression and BPD (with levels similar to those of the diffused identity class), but tended to improve in psychological functioning over time. Opposite findings were obtained for adolescents in the identity regression class, who indicated high baseline levels of self-esteem and resilience, and low baseline levels of symptoms of depression and BPD (with levels similar to those of the adaptive identity class), but tended to worsen in psychological functioning over time. Although focusing on another indicator of psychosocial functioning (i.e., family functioning), Schwartz et al. (9) yielded similar findings. Specifically, adolescents who decreased in identity confusion (i.e., identity progression) tended to show the greatest improvements in psychosocial functioning, whereas adolescents who increased in identity confusion (i.e., identity regression) tended to show a worsening in psychosocial functioning over time. Although our study design does not allow to make developmental inferences, these findings seem to support leading theories by indicating that improvements in identity functioning may lay the foundation for psychological well-being, whereas setbacks in identity functioning may enlarge the risk of psychopathology (2, 26, 43). Differently, de Moor et al. (15) did not find clear significant differences in substance abuse among stable, progressive, and regressive identity classes in adolescence.

In summary, the present study seems to confirm that having or developing towards a consolidated sense of identity represents an important source for positive psychological functioning. Specifically, (regaining) healthy identity functioning seems to coexist with stable high or increasing levels of self-esteem and resilience, and stable low or decreasing levels of symptoms of depression and BPD. In contrast, suffering from stable or increasing levels of disturbed identity and/or lack of identity seems to coexist with stable low or diminishing positive psychological functioning, as well as stable high or increasing levels of psychopathology.

Results from the present study should be interpreted in light of some limitations. First, we used self-report measures to assess all variables. Although self-report data are assumed to provide a relatively true picture of identity functioning, the exclusive use of quantitative self-report data might have led to inflated correlations among variables and reporting biases (87). In future research, we could consider adopting a multi-method (e.g., both quantitative and narrative data on identity) and/or multi-informant study design to increase the reliability of our findings. Second, despite its three-wave longitudinal design, the present study considered a relatively short time span of 2 years and focused exclusively on adolescents. Future studies might include more measurement points to adopt an accelerated or cohort-sequential study design in which both linear and *non-linear* trends in identity development can be charted throughout adolescence and in which differences in identity development across early, mid-, and late adolescence can be taken into account. Moreover, future studies might also attend to identity development and psychological functioning in emerging and young adults, as important changes in identity formation still occur during this transitional life stage (8, 71, 88). Third, although research has mainly studied identity development over large time intervals (e.g., 6 months or 1 year), emerging research evidences daily dynamics in identity formation (66, 89, 90), which should encourage researchers to investigate day-to-day trends in identity development and how they may feed into daily psychosocial functioning. Fourth, our findings are dependent upon our use of the SCIM. While this instrument advances our understanding of both adaptive and disturbed dimensions of identity development, the dimensions of the SCIM differ from those considered in previous research (e.g., identity processes specific to the U-MICS or the Dimensions of Identity Developmental Scale, and identity synthesis and confusion dimensions; 7, 41, 91) as the SCIM assesses aspects of personal *and* social identity functioning. Although insightful, it is difficult to compare our results with those of previous research. Yet, our findings could encourage researchers to study the interplay of personal and social identity formation, since their development may be largely enmeshed. For instance, research has indicated that, throughout adolescence, social identifications (e.g., with classmates) seem to be a source for subsequent personal identity formation (92). Finally, the findings obtained in our study apply to Western (specifically, Flemish) adolescents and cannot be generalized to other non-Caucasian or more diverse samples. Future research could investigate our research questions among adolescents of both Western and non-Western cultures to advance our understanding of cross-cultural variations in identity development and links with psychological functioning. As the development of an integrated and autonomous sense of identity may be more valued in individualistic countries (such as Belgium) than in collectivistic countries (such as Japan), identity development and its associations with psychological well-being may vary (93, 94).

Notwithstanding these limitations, the present results emphasize the importance of both adaptive and disturbed identity dimensions in adolescence. Our findings indicate that adolescents who struggle with emerging or lasting identity disturbance and/or lack of identity have an increased risk of experiencing increasing or enduring mental health problems such as low levels of self-esteem and resilience, and high levels of depressive symptoms and BPD features. Alternatively, adolescents who experience a stable or increasing sense of identity consolidation seem to profit from stable high or increasing levels of psychological well-being as well as stable low and decreasing levels of psychological dysfunctioning. Altogether, our results underscore the importance of supporting healthy identity development and treating identity dysfunctioning to promote mental health in adolescence.

Although having identity issues must not be considered a clinical problem in young individuals, their distressing consequences should not be minimalized or trivialized. Rather, adolescents’ identity development should be fostered by parents, teachers, and (if necessary) psychologists. In a first step, caregiving figures can support adolescents in coping with their transforming identity by normalizing feelings of identity confusion, without dismissing them, and by underscoring their adaptive function as they essentially stimulate youngsters to explore new life paths and strive for personal identity (2, 95). In addition, parents can potentially mitigate (or even prevent) identity issues in their children by providing them with a safe and (autonomy) supportive environment (96). Finally, should adolescents be challenged with persistent identity formation problems and be at risk of developing psychopathology, they should be able to receive psychotherapy that includes identity-specific modules prioritizing the treatment of problems related to self and identity. Effective evidence-based interventions for treating both symptom and personality disorders, consisting of (among others) Mentalization-Based Treatment (MBT; 97), Dialectical Behavior Therapy (DBT; 98), and Transference-Focused Psychotherapy (TFP; 99), have been adapted for use in adolescents. All of these interventions assume that psychopathology results in part from a poorly developed, unstable, or negative sense of self and others, and may thus be effective in bolstering identity development in adolescence.

## Data availability statement

The raw data supporting the conclusions of this article will be made available by the authors, without undue reservation.

## Ethics statement

The present study was approved by the ethical committee (SMEC) of the Faculty of Psychology and Educational Sciences of KU Leuven. Written informed consent to participate in this study was provided by the participants’ legal guardian/next of kin.

## Author contributions

AB, LC, and KL contributed to the design of the study. AB wrote the first draft of the manuscript. AB and KR analyzed the data. AB and TBu collected the data. All authors contributed to the article and approved the submitted version.

## Funding

The present study was funded by grant G062117N from the fund for Scientific Research in Flanders (FWO).

## Conflict of interest

The authors declare that the research was conducted in the absence of any commercial or financial relationships that could be construed as a potential conflict of interest.

## Publisher’s note

All claims expressed in this article are solely those of the authors and do not necessarily represent those of their affiliated organizations, or those of the publisher, the editors and the reviewers. Any product that may be evaluated in this article, or claim that may be made by its manufacturer, is not guaranteed or endorsed by the publisher.
