# Supplementary material for: Trajectories of adaptive and disturbed identity dimensions in adolescence: developmental associations with self-esteem, resilience, symptoms of depression, and borderline personality disorder features
Source: Front Psychiatry. 2023 Apr 24;14:1125812. doi: 10.3389/fpsyt.2023.1125812 (PMC10165116; doi:10.3389/fpsyt.2023.1125812)
Supplement: Supplementary file 1 [file Table_1.DOCX]

**Supplementary Material**

Table 1. *Fit indices for different Growth Mixture Models for adolescent girls and boys who completed the BPFS-C.*

|  | | | | Trajectory class prevalence (%) | | | | | |
| --- | --- | --- | --- | --- | --- | --- | --- | --- | --- |
| Solution | BIC | E | b-LRT | 1 | 2 | 3 | 4 | 5 | 6 |
| Total subgroup | | | | | | | | | |
| 1 | 31299.792 |  |  | 100.00 |  |  |  |  |  |
| 2 | 30880.880 | .87 | *p* < .001 | 22.66 | 77.34 |  |  |  |  |
| 3 | 30709.229 | .88 | *p* < .001 | 10.91 | 68.12 | 20.97 |  |  |  |
| **4** | **30634.378** | **.85** | *p* < .001 | **67.34** | **13.70** | **9.81** | **9.16** |  |  |
| 5 | 30595.229 | .84 | *p* < .001 | 6.75 | 13.90 | 8.25 | 62.34 | 8.77 |  |
| 6 | 30524.825 | .85 | *p* < .001 | 4.55 | 14.61 | 59.29 | 6.17 | 6.49 | 8.90 |
| Girls | | | |  | | | | | |
| 1 | 19206.009 |  |  | 100.00 |  |  |  |  |  |
| 2 | 19036.851 | .83 | *p* < .001 | 71.49 | 28.51 |  |  |  |  |
| 3 | 18981.053 | .85 | *p* < .001 | 25.77 | 13.82 | 60.42 |  |  |  |
| **4** | **18974.874** | **.81** | ***p* < .001** | **10.31** | **13.60** | **60.31** | **15.79** |  |  |
| 5 | 18975.498 | .82 | *p* < .001 | 16.01 | 6.36 | 8.99 | 9.65 | 58.99 |  |
| 6 | 18968.152 | .81 | *p* < .001 | 8.55 | 12.06 | 9.10 | 52.63 | 4.83 | 12.83 |
| Boys | | | | | | | | | |
| 1 | 11968.971 |  |  | 100.00 |  |  |  |  |  |
| 2 | 11761.760 | .92 | *p* < .001 | 16.56 | 83.44 |  |  |  |  |
| 3 | 11707.104 | .91 | *p* < .001 | 6.37 | 17.68 | 75.95 |  |  |  |
| **4** | **11639.104** | **.89** | ***p* < .001** | **9.24** | **15.13** | **71.34** | **4.30** |  |  |
| 5 | 11592.541 | .90 | *p* < .001 | 7.80 | 4.14 | 68.15 | 3.18 | 16.72 |  |
| 6 | 11586.155 | .89 | *p* < .001 | 7.17 | 3.82 | 8.28 | 12.58 | 3.03 | 65.13 |

*Note.* The selected class-solutions are presented in bold.
